# Volumetric Optical Imaging and Quantitative Analysis of Age-Related Changes in Anterior Human Vitreous

**DOI:** 10.1167/iovs.62.4.31

**Published:** 2021-04-30

**Authors:** Daniel Ruminski, J. Sebag, Raúl Duarte Toledo, Alfonso Jiménez-Villar, Jan K. Nowak, Silvestre Manzanera, Pablo Artal, Ireneusz Grulkowski

**Affiliations:** 1 Institute of Physics, Faculty of Physics, Astronomy and Informatics, Nicolaus Copernicus University, Torun, Poland; 2 VMR Institute for Vitreous Macula Retina, Huntington Beach, California, United States; 3 Laboratorio de Óptica, Instituto Universitario de Investigación en Óptica y Nanofísica, Universidad de Murcia, Campus de Espinardo, Murcia, Spain; 4 Department of Pediatric Gastroenterology and Metabolic Diseases, Poznan University of Medical Sciences, Poznan, Poland

**Keywords:** vitreous, optical coherence tomography (OCT), aging, contrast sensitivity, opacification

## Abstract

**Purpose:**

The purpose of this study was to characterize age-related changes in anterior human vitreous with 3-D swept source optical coherence tomography (SS-OCT) and evaluate associations with axial length (AL) and contrast sensitivity function (CSF).

**Methods:**

There were 49 phakic eyes in 49 patients (40.0 ± 19.3 years) had 3-D volumetric scanning of the lens and retrolental vitreous with SS-OCT at 1050 nm. OCT-derived indices of vitreous optical density (VOD), vitreous opacification ratio (VOR), and lens optical density (LOD) were correlated with AL and double-pass assessment of retinal point spread function (Objective Scatter Index [OSI]). CSF was measured using an adaptive-optics visual simulator (area under log-log contrast sensitivity function [AULCSF]).

**Results:**

Vitreous SS-OCT detected gel vitreous, liquefied lacunae, Berger's space, retrolental laminae, and fibrous opacifications. VOD, VOR, and LOD showed high reproducibility (intraclass correlation coefficients 0.968, 0.975, and 0.998, respectively). VOD was highly correlated with VOR (Pearson's R = 0.96, *P* < 0.000001). VOD, VOR, and LOD correlated with age (R = 0.48, 0.58, and 0.85, *P* < 0.001 for each). VOR and LOD correlated with OSI (R = 0.36, *P* = 0.0094, and R = 0.36, *P* = 0.0096, respectively). VOR correlated negatively with AULCSF (R = −0.53, *P* < 0.00009), which was related to OSI. Myopic eyes had higher OSI than nonmyopic eyes (*P* = 0.0121), consistent with correlation between OSI and AL (R = 0.37, *P* = 0.0091). Multivariable regression confirmed these findings.

**Conclusions:**

SS-OCT visualized microstructural features of anterior human vitreous, where opacification is associated with increased light scattering and CSF degradation. SS-OCT enables high-resolution optical evaluation of vitreous opacities.

The vitreous body is a hydrophilic gel located between the crystalline lens and retina, constituting the largest volumetric component of the human eye. In youth, vitreous is optically transparent, composed mostly of water, with a very low concentration of macromolecules (collagen and hyaluronan) providing structural support to the globe and stability during rapid eye movements and strenuous physical activity.^1^ Optical transparency of the vitreous body is the result of specific organization of these molecular components. As a repository for anti-oxidants and a pathway for nutrients utilized by the crystalline lens, ciliary body, and retina, vitreous is important in ocular physiology, especially as it relates to intra-ocular oxygen metabolism.^2^^,^^3^

Aging of the vitreous body is characterized by fibrous degeneration with gel liquefaction.^4^^,^^5^ Fibrous vitreous liquefaction results from molecular changes causing dissociation of collagen from hyaluronan, forming liquid vitreous (water molecules bound to hyaluronan) and cross-linking of collagen into visible fibers.^6^^,^^7^ In the presence of myopia, these changes appear earlier in life, and in direct proportion to the degree of axial myopia.^8^^,^^9^ Consequently, vitreous structure becomes more heterogeneous with the formation of liquefied pockets called lacunae and aggregation of collagen fibrils into larger fibers that present clinically as opacities, which cause the disturbing visual phenomenon of “floaters.”^4^^,^^10^ Fibrous vitreous liquefaction not only introduces structural heterogeneity, but also promotes posterior vitreous detachment (PVD), a common cause of increased intra-ocular light scattering^11^ and floaters, at times with degradation of contrast sensitivity function.^11^^–^^13^ At this point, the condition attains clinical significance and is called “vision degrading myodesopsia.”^14^ Apart from age, other factors contributing to fibrous vitreous liquefaction/degeneration include diabetic vitreopathy,^15^ inflammation,^16^ and myopic vitreopathy,^9^ the latter associated with increased incidence of PVD. Persistent vitreo-retinal adhesion can induce anomalous PVD resulting in vitreo-macular traction syndrome, retinal tears, and detachment,^7^ at times splitting of the posterior vitreous cortex (vitreoschisis),^17^ contributing to premacular membranes with macular pucker and macular hole formation.^5^^,^^14^^,^^18^ Anterior vitreous opacification can also occur, particularly after surgical interventions of the posterior lens.^19^^–^^21^ What is not known, however, is whether anterior vitreous changes are associated with central/posterior fibrous liquefaction/degeneration and vision degrading myodesopsia.

The optical properties of the vitreous body that make it transparent, render vitreous as one of the most challenging of all ocular structures to visualize. Attempts to date were mostly limited to slit-lamp biomicroscopy, ophthalmoscopy, B-scan ultrasonography, and magnetic resonance imaging.^10^^,^^22^^–^^28^ Optical coherence tomography (OCT) is an imaging modality that has become a standard method in the diagnosis of retinal disorders.^29^ Current clinical OCT technology uses either a broadband light source and a spectrometer (spectral-domain OCT [SD-OCT]) or a wavelength-tunable light source and a high-speed point detector (swept source OCT [SS-OCT]). Due to the high sensitivity of OCT compared with other optical imaging technologies, OCT has demonstrated effective visualization of the posterior vitreous,^30^^–^^32^ but not the central or anterior vitreous. The approach called enhanced vitreous imaging (EVI) enables better insight into vitreous degeneration and posterior vitreous detachment,^33^^–^^37^ but still has limitations in imaging the entire vitreous body. Recent advances in tunable lasers and high-speed electronics enable imaging of the entire length of the eye.^38^ To date, OCT imaging of the anterior vitreous has been limited to studies of Berger's space in cases associated with posterior lens capsule surgery (e.g. capsulorhexis and capsulotomy).^21^^,^^39^^,^^40^

The aim of this study is to image the retrolental (anterior) vitreous with 3-D high-resolution SS-OCT, and to study the associations of those changes with age, axial length, and vision, specifically contrast sensitivity function. We describe micro- and macroscopic structural features of aging retrolental vitreous as well as the vitreo-lenticular interface, and perform quantitative analysis of retrolental vitreous opacification in relation to the loss of transparency of the crystalline lens. Furthermore, we investigate associations between intra-ocular light scattering in the crystalline lens and in the retrolental vitreous with the contrast sensitivity function.

## Methods

This cross-sectional observational study was approved by the Institutional Ethics Committees at the University of Murcia (Murcia, Spain) and at the Nicolaus Copernicus University (Torun, Poland) and adhered to the tenets of the Declaration of Helsinki. Each participant was informed about the nature of the study, and informed consent was obtained prior to the measurements. In the cases of enrolled children, written informed consent was given by the parents or guardians. The protocol excluded subjects with previous eye surgery. No eye dilation was induced during the measurements, which were conducted under dimmed room lighting to avoid possible pupil constriction.

### Ocular Biometry, Eye Aberrations and Measurement of Intraocular Scattering

Ocular biometry was performed with optical biometer based on optical low-coherence reflectometry (Lenstar, Haag-Streit AG, Switzerland). The wavefront aberrations of the eye and contrast sensitivity measurements were obtained with a clinical adaptive optics visual simulator (VAO, Voptica S.L., Spain).^41^^,^^42^ Objective scatter index (OSI) was determined from double-pass retinal point spread function (PSF) images (OQAS HD Analyzer, Visiometrics, Spain).^43^^,^^44^

### OCT Imaging

**Figure 1.**
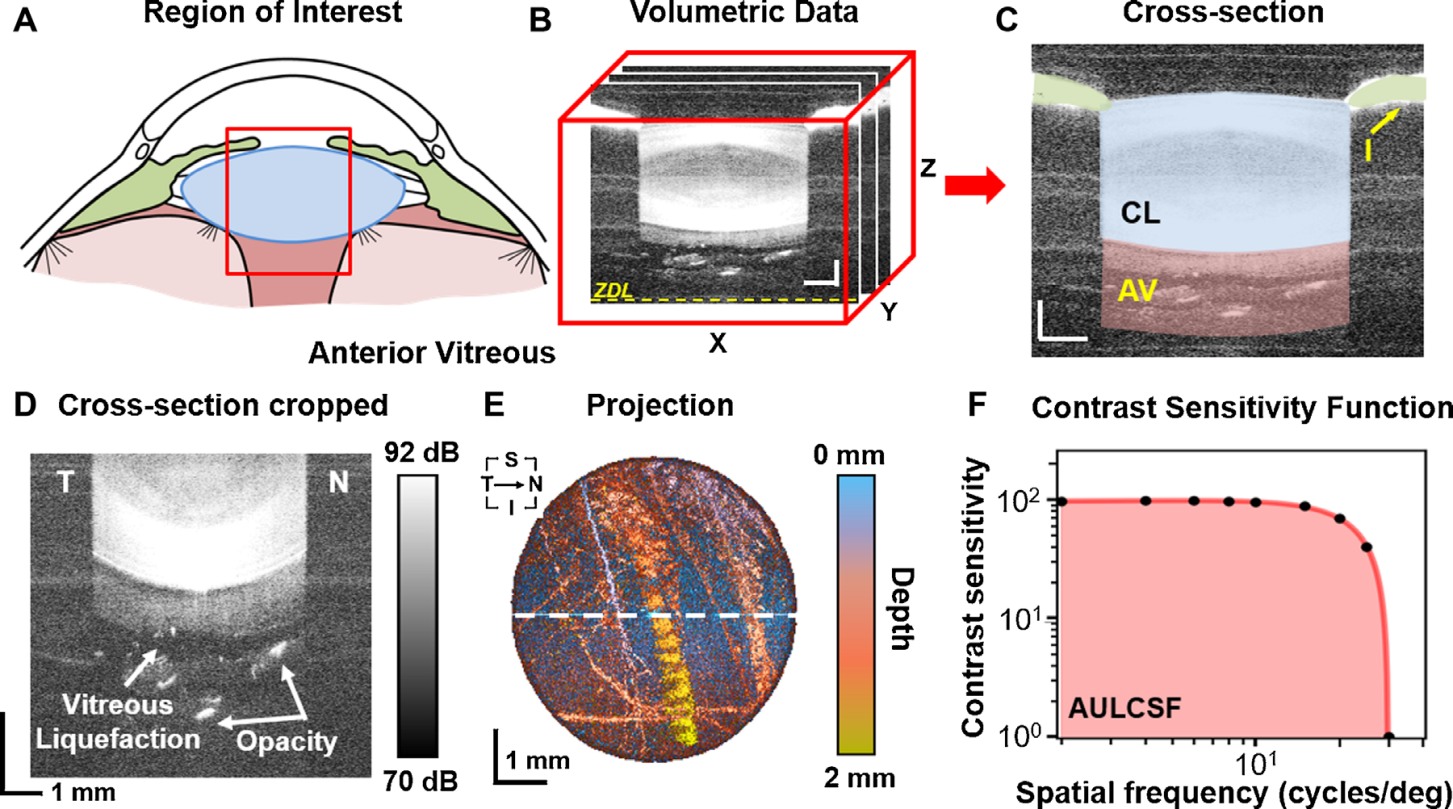
Imaging/quantitative analysis of retrolental vitreous and assessment of contrast sensitivity. (**A**) Schematic representation of the region of interest for the study. The *red rectangle* indicates the region evaluated with SS-OCT that includes the iris, the crystalline lens, and the retrolental vitreous. (**B**) Example of a cropped volumetric OCT dataset 8 × 8 × 8 mm^3^ (300 A-scans × 300 B-scans × 1200 pixels): ZDL = zero delay line. (**C**) Central cross-sectional image with colored morphological structures: CL = crystalline lens, I = iris, AV = anterior vitreous. Scale bar = 1 mm. (**D**) Zoomed cross-section of the anterior vitreous with hypo-reflective liquefied region. (**E**) En face maximum intensity projection (MIP) of the anterior vitreous from 2-mm thick slab behind the posterior lens capsule. The depth of the signal was color coded. T = temporal, N = nasal, S = superior, I = inferior. Scale bar = 1 mm. (**F**) Contrast sensitivity function presented in log-log scale. AULCSF = area under the log-log contrast sensitivity function highlighted in *red*. This was from a 48 year old subject, OD.

OCT imaging was performed with a prototype SS-OCT instrument operating at the central wavelength of 1050 nm (bandwidth 110 nm) and at the sweep rate of 50 kHz.^45^ The power of the probing light beam at the cornea (1.9 mW) did not exceed American National Standard Institute (ANSI) standards. The measured axial and transverse resolutions in air were 9 μm and 43 μm, respectively. The light source and optical detection characteristics enabled minimization of the signal drop with depth. The interface of the system was designed to achieve high sensitivity of 103 dB and low sensitivity drop with depth (−6 dB at 11 mm and −20 dB at 22 mm), which was crucial to detect photons scattered by vitreous. Similar to enhanced depth imaging for choroidal visualization in standard SD-OCT systems,^46^ the objective lens of the SS-OCT instrument was moved closer to the eye so that the focal plane of the scanning beam was positioned slightly behind the posterior surface of the crystalline lens to maximize light collection efficiency from the anterior vitreous (Fig. 1A). Moreover, we set the length of the reference arm to place the vitreous close to zero delay line (ZDL) in OCT images (Fig. 1B). Thus, optimization of instrument speed, detection system interface, focal plane, and tuning of the reference arm length augmented instrument sensitivity to allow for high-resolution in vivo vitreous imaging. The signal in OCT cross-sections was displayed in logarithmic scale to account for the large dynamic range of light backscattered from anterior segment structures and vitreous. In particular, the contrast window was adjusted to enhance vitreous visualization in the same way for all OCT images.^33^

### OCT Data Postprocessing and Quantitative Analysis

Three SS-OCT data sets were acquired for each subject's eyes. The 3-D volumetric data sets of the anterior segment and retrolental vitreous contained 300 × 300 A-scans and covered the area of 8 × 8 mm^2^ of the pupil plane. Acquisition time of the single cubic scan was less than 2 seconds. Motion correction of involuntary eye movements was performed in axial and transverse directions in postprocessing. First, the axial eye motion was detected by delineating the posterior lens capsule interface using the Sobel filter of Gaussian blurred cross-sections. Differences of detected motion-corrupted interface and best paraboloid fit were used to correct for axial eye motion. Next, transverse motion correction was based on masking the pupil area and shifting the B-scans to obtain circular shape of the pupil. Subsequently, crystalline lens interfaces were segmented in a motion-free data set to define two regions of interest: crystalline lens (the space between the anterior and posterior capsules of the lens, blue in Fig. 1C) and anterior vitreous (the 2 mm thick slab behind the posterior lens capsule, pink in Fig. 1C). OCT signals (in linear scale) were averaged in both regions of interest to determine vitreous optical density (VOD) and lens optical density (LOD). Zoomed OCT images demonstrated vitreous opacifications (Fig. 1D). Accordingly, the region of anterior vitreous was also thresholded (>78 dB) to yield hyper-reflective (hyperlucent) regions corresponding to opacities. The total number of thresholded voxels normalized to the total volume of the anterior vitreous defined vitreous opacification ratio (VOR). This parameter yields values between zero (no opacification above set threshold) and one (totally opacified). All proposed parameters were calculated automatically from 3-D OCT data sets using custom designed software prepared in Python programming language, and three measurements from each eye were averaged. Furthermore, the anterior vitreous slab was used to generate maximum intensity projection, in which the depth of the maximum intensity voxel below posterior lens interface was color coded (Fig. 1E).

### Assessment of Contrast Sensitivity Function

The contrast sensitivity function (CSF) was determined based on the fringes with a calibrated contrast presented to the subject by a previously described visual simulator.^47^ The CSF was plotted versus target spatial frequency in log-log scale (Fig. 1F), and the area under the log-log contrast sensitivity function (AULCSF) was calculated.

### Statistical Analyses

Although both eyes were measured in each subject, one eye in each subject was randomly selected for statistical analyses to avoid the bias of the bilateral eye correlation. Statistical analysis included reproducibility assessment, correlation analysis, and regression. The reproducibility of defined parameters (i.e. VOD, VOR, and LOD) was evaluated using one-way analysis of variance with a random-effects model and expressed with intraclass correlation coefficient (ICC). The ICC was tested for statistical significance and 95% confidence interval (95% CI) of ICC was determined. Pearson's R correlation coefficient was calculated for extracted parameters and statistical significance (*P* value) threshold was set at α = 0.05.

The data were also stratified by axial length (AL), and two subgroups were selected to determine any differences in measured and calculated parameters: nonmyopic eyes (AL < 23 mm) and myopic eyes (AL > 25 mm). The normality of distribution was tested using Shapiro-Wilk normality test. The differences between normally distributed variables were analyzed with the *t*-test for independent samples. When the data were not normally distributed, the differences were tested using the Mann-Whitney test.

To identify independent associations between measured and calculated parameters and to compensate for confounding, forward stepwise linear regression analysis was conducted with R 4.0.2 (R Foundation, Vienna, Austria). Variables with non-normal distributions (the Shapiro-Wilk test and histogram inspection) were transformed using the Tukey's ladder of powers (*rcompanion* version 2.3.25). Linear models were fitted with forward stepwise variable selection using *MASS* 7.3–51.6 and β coefficients were obtained with *lm.beta* 1.5-1. Adjusted R-squared (aR^2^) were also calculated. To minimize collinearity, only the following variables were included: VOD, VOR, LOD, age, AL, and AULCSF.

## Results

This study included 49 phakic eyes of 49 subjects (25 men and 24 women; mean age = 40.0 ± 19.3 years, age range = 9–78 years). The mean sphere was −1.0 ± 2.2 D (range = −9 D to +3.75 D), and the mean cylinder was −0.60 ± 0.55 D (range = −2 D to 0 D). The mean axial eye length was 23.90 ± 1.05 mm (range = 22.06 mm to 26.16 mm).

**Figure 2.**
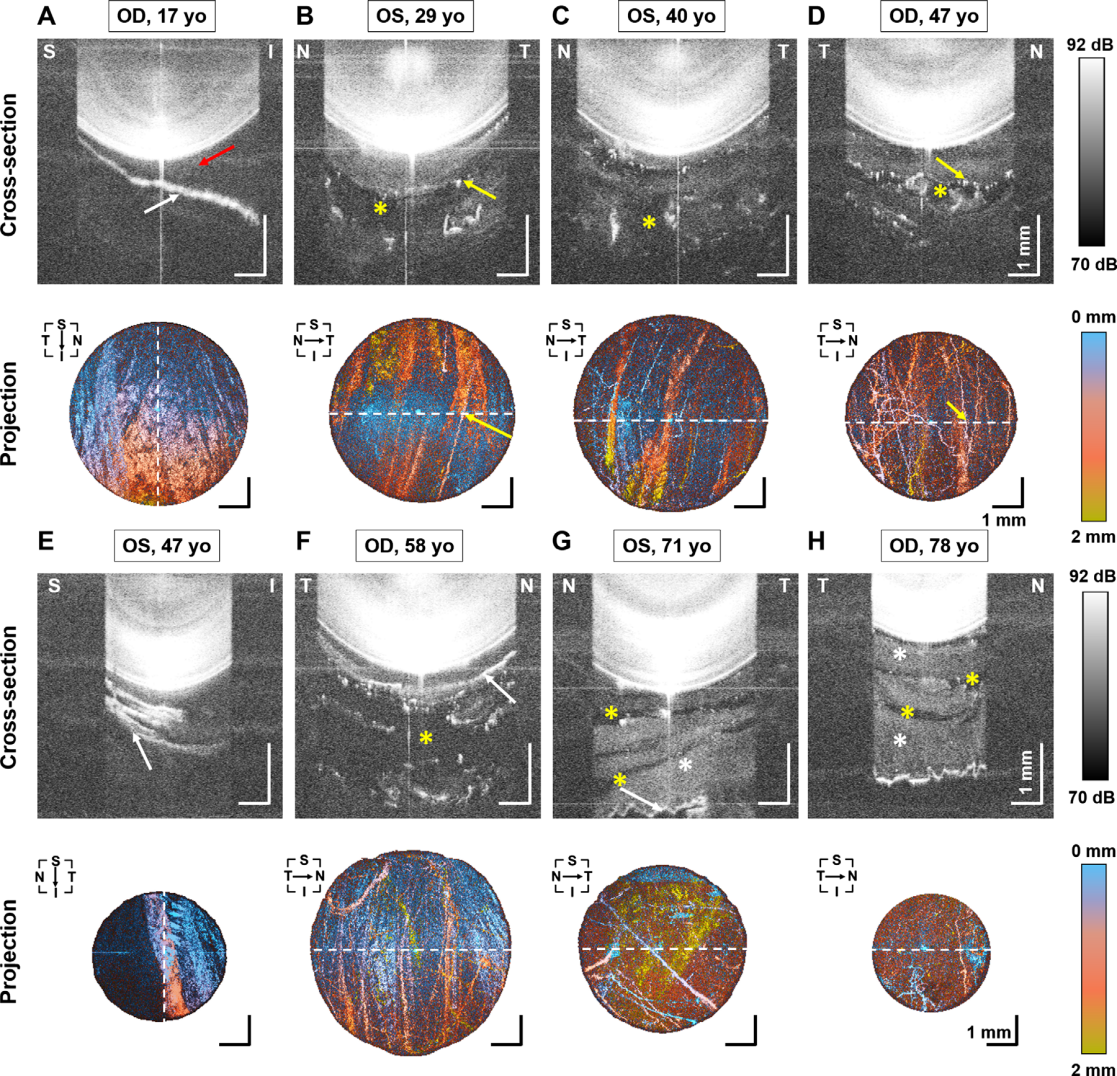
Characteristic features of retrolental vitreous in 3-D SS-OCT images. Cross-sectional images and projection images of the anterior vitreous. (**A**) From a 17 year old subject, OD. (**B**) From a 29 year old subject, OS. (**C**) From a 40 year old subject, OS. (**D**) From a 47 year old subject, OD. (**E**) From a 47 year old subject, OS. (**F**) From a 58 year old subject, OD. (**G**) From a 71 year old subject, OS. (**H**) From a 78 year old subject, OD. *Dashed lines* represent section direction. *White arrows* indicate laminar structures. The *red arrow* indicates the Berger's space. The *yellow arrows* denote fiber-like opacities. The *yellow asterisks* indicate lacunae with liquid vitreous. The *white asterisks* correspond to the gel vitreous. T = temporal, N = nasal, S = superior, I = inferior. Scale bar = 1 mm.

SS-OCT successfully obtained volumetric scans of the entire anterior segment, including retrolental vitreous in all subjects. Figure 2 demonstrates cross-sections and corresponding vitreous projection images of the anterior vitreous in subjects of different ages. A number of anterior vitreous microstructural features could be distinguished on OCT imaging. The signal above the background can be referred to as gel vitreous, which results from the presence of light-scattering vitreous macromolecules (white asterisks in Figs. 2G, 2H). Laminar structures were visible in 33 out of 49 eyes (67%). OCT images revealed that the laminae had different shapes: flat, plicated, or irregularly corrugated (white arrows in Figs. 2A, 2E–2H, respectively), and do not occupy the entire pupil area. In the case presented in Figure 2A, the space between the crystalline lens and the lamina forms Berger's space (red arrow in Fig. 2A). Moreover, numerous hyper-reflective spots in OCT cross-sectional images represent opacities in the retrolental vitreous (e.g. yellow arrows in Figs. 2B, 2D). Better insight into opacity architecture can be obtained from projection images, which reveal that those “spots” are actually networks of fibers. In some cases, the fibrous opacifications surround extremely hyporeflective zones (with the signal at the level of background), which can be identified as the lacunae of liquefied vitreous (yellow asterisks in Figs. 2B–D, 2F–H).

Quantitative analysis of the OCT data was based on calculating three indices that assess scattering in vitreous (VOD) and the crystalline lens (LOD), as well as the degree of vitreous opacification (VOR). ICCs calculated for VOD, VOR, and LOD were 0.968 (95% CI = 0.950–0.981), 0.975 (95% CI = 0.960–0.985), and 0.998 (95% CI = 0.997–0.999), respectively. Statistical significance of ICC was obtained for all values of ICC (*P* < 10^−64^).

**Figure 3.**
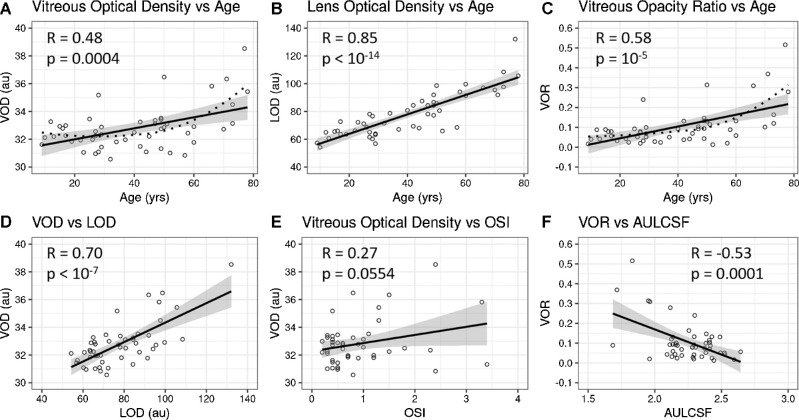
Correlation plots of vitreous and lens densities with age and each other. There are statistically significant positive correlations between age and vitreous density (VOD) (**A**), lens optical density (LOD) (**B**), and vitreous opacity ration (VOR) (**C**); **A** and **C** with locally estimated scatterplot smoothing (LOESS) with a span of 1 to obtain curves optimally fitting the data (*dotted curves*). Vitreous density also correlated positively with lens density (**D**), and objective scatter index (OSI) (**E**), as well as negatively correlated with contrast sensitivity function (AULCSF) (**F**), meaning that with increasing vitreous density (VOR) there was degradation of contrast sensitivity function. 95% confidence interval bands are shown in *grey*.

The images in Figure 2 are ordered according to subject age. Visual inspection of the images shows increasing light scattering in both the anterior vitreous and the crystalline lens with age. The results of correlation analysis are summarized in Table 1, and selected correlation plots are shown in Figure 3.

**Table 1.** Pearson's Correlation and *P* Value Between the Studied Parameters

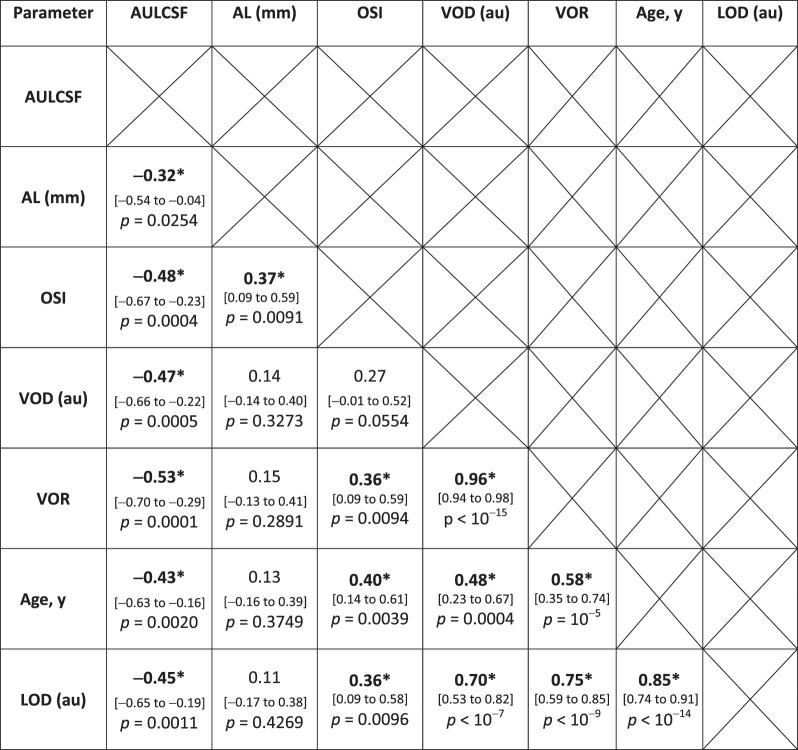

The 95% confidence intervals are reported in the square brackets.

AULCSF, area under log-log contrast sensitivity function; AL, axial eye length; OSI, Objective Scatter Index; VOD, vitreous optical density; VOR, vitreous opacification ratio; LOD, lens optical density.

Asterisks indicate statistical significance.

Statistically significant correlations were found between OCT-based parameters (VOR, VOD, or LOD) and age (R = 0.58, 0.48, and 0.85, respectively, *P* < 0.001), meaning that structural changes within the crystalline lens and anterior vitreous are strongly related with age (Figs. 3A–C). However, the plots in Figures 3A and 3C show that a nonlinear regression (applied as locally estimated scatterplot smoothing method [LOESS]) better describes this relation (as indicated by residual standard error: in Fig. 3A 1.415 for linear model versus 1.297 for LOESS; in Fig. 3C 0.083 for linear model versus 0.076 for LOESS). The mean signal of the crystalline lens (LOD) was usually 2 to 3 times higher than that of the anterior vitreous (VOD). In addition, the VOD correlated very strongly with the VOR (R = 0.96, *P* < 10^−6^). The results also indicate a strong correlation between OCT signals in the lens (LOD) and the vitreous (VOD or VOR), with R = 0.70, *P* < 10^−5^ (Fig. 3D) and R = 0.75, *P* < 10^−5^, respectively. It is important to note that we detected no correlation of AL with either VOD or VOR (R = 0.14, *P* = 0.33 and R = 0.15, *P* = 0.29, respectively). The OSI correlated with VOR (R = 0.36, *P* = 0.0094) and LOD (R = 0.36, *P* = 0.0096) but not with VOD (R = 0.27, *P* = 0.0554; Fig. 3E).

The AULCSF demonstrates rapid CSF degradation with spatial frequency (lower AULCSF indicates CSF degradation). The results revealed that AULCSF negatively correlated with all other parameters and that association with vitreous opacification (VOR) was the most pronounced (R = −0.53, *P* = 9 × 10^−5^), as shown in Figure 3F.

Myopic eyes had higher OSI than nonmyopic eyes (*P* = 0.0121), which is consistent with observed correlation between OSI and AL in the entire study population (R = 0.37, *P* = 0.0091; see Table 1). No statistically significant differences in VOD, VOR, LOD, or AULCSF were detected between myopic and nonmyopic subgroups (Table 2), however, this may be due to the small sample sizes (*n* = 10 nonmyopic and 6 myopic subjects) in these subgroup analyses.

**Table 2.** Assessment of Differences of Measured Parameters in Myopic and Nonmyopic Eyes

Parameter	Nonmyopic Eyes AL < 23 mm (*n* = 10)	Myopic Eyes AL > 25 mm (*n* = 6)	*P* Value
Age, y	32.4 ± 22.1	41.5 ± 12.2	0.3721
Sphere (D)	0.72 ± 1.36	-4.75 ± 1.87	**0.0017** ^*^
AL (mm)	22.29 (22.11–22.85)	25.72 ± 0.43	**0.0014** ^*^
VOD (au)	32.05 ± 0.71	32.71 ± 2.04	0.3571
VOR	0.06 ± 0.04	0.083 (0.031–0.092)	0.5622
LOD (au)	71.1 ± 14.5	76.3 ± 14.4	0.4970
OSI	0.4 (0.3–0.4)	1.23 ± 0.80	**0.0121** ^*^
AULCSF	2.36 ± 0.17	2.17 ± 0.20	0.0619

Data are given as mean ± standard deviation or median (Q1–Q3). AL, axial eye length; VOD, vitreous optical density; VOR, vitreous opacification ratio; LOD, lens optical density; OSI, Objective Scatter Index; AULCSF, area under log-log contrast sensitivity function.

* Asterisk indicates statistically significant difference.

Regression analysis was performed to control for confounding effects and assess independent variables. Multivariable regression confirmed a strong independent association between age and lens opacification (LOD; adjusted R^2^ = 0.77, *P* = 9.10 × 10^−13^, and age β = 0.64, *P* = 2.66 × 10^−8^). Vitreous opacification (VOD) was linked to lens opacification (LOD) alone and not age (VOD aR^2^ = 0.39, *P* = 7.76 × 10^−5^, LOD β = 0.88, *P* = 1.37 × 10^−4^) and a similar result was obtained for vitreous opacification ratio (VOR aR^2^ = 0.48, *P* = 3.49 × 10^−6^, and LOD β = 0.84, *P* = 8.33 × 10^−5^). However, a model including untransformed data suggested that age might also independently associate with anterior vitreous opacification (VOD). Furthermore, AULCSF was linked to OSI (AULCSF aR^2^ = 0.30, *P* = 1.76 × 10^−3^, and OSI β = −0.34, *P* = 3.11 × 10^−2^).

## Discussion and Conclusions

Extensively detailed, in vivo SS-OCT imaging of retrolental (anterior) vitreous uncovered the rich and underexplored landscape of its morphology. We demonstrate age-related structural alterations of not only the anterior vitreous, but also the vitreo-lenticular interface, and quantify positive correlation between the vitreous and lens opacifications. Previous studies using dark-field slit microscopy qualitatively characterized age-related changes in vitreous structure,^4^ whereas ultrasonography established this relationship quantitatively.^13^ To date, OCT imaging of the anterior vitreous has been confined to the identification of Berger's space in a series of case reports,^20^^,^^21^^,^^39^^,^^40^ and was never used to perform quantitative analysis of vitreous structure here or anywhere in the vitreous body. Although alterations in the posterior vitreous and at the vitreo-retinal interface can be imaged by OCT, even a change as profound as posterior vitreous detachment cannot be accurately diagnosed with conventional OCT imaging.^48^ Furthermore, the study of the anterior vitreous was previously hindered by technological problems, which we resolved in this investigation.

In the present study, several features of OCT were used to overcome limitations of other diagnostic methods to assess the anterior vitreous. First, slit-lamp examination only provides a 2-D cross-sectional view, and not a 3-D scan. Second, the near infrared light source used in SS-OCT instrumentation (∼1 μm) does not induce pupillary constriction and allows scanning large pupil areas at high resolution without mydriatic drops. Third, whereas B-scan ultrasonography has advantages over OCT in terms of the imaging range that permits investigation of the full depth of the vitreous body, ultrasound has limited resolution, and the measurement requires contact of the instrument with the eye, or at least the eyelid. Volumetric 3-D SS-OCT imaging supports different data visualization strategies, such as rendering, cross-sections, and projections, which enhances diagnostic utility. Moreover, the OCT data can be further processed to perform objective quantification of structural density.

Our research revealed that SS-OCT can visualize micro-structural details, including Berger's space and diverse laminar or fibrous structures. The anterior vitreous body was optically homogenous in younger age and became progressively more heterogeneous above 60 years of age. Gel and liquefied fractions of the vitreous could be easily distinguished in OCT images. Macromolecules (primarily collagen, but possibly also hyaluronan conjugated with other extracellular matrix components) scattered light in gel vitreous, whereas liquefied vitreous was observed as hypo-reflective lacunae in OCT images. Three-dimensional high-resolution imaging also enabled comprehensive identification of age-related opacification in the retrolental vitreous. Projection images (see Fig. 2) revealed characteristic fibrous (linear) opacifications at the borders between gel and liquid vitreous (lacunae), which might be associated with collagen aggregates. Identical forms of opacities were previously observed in the posterior vitreous.^49^ Laminar structures (see Figs. 2A, 2E–2H) may be remnants of the anterior embryonic hyaloid vasculature, which forms an anastomosis with the tunica vasculosa lentis. Another possibility is that the multi-laminar appearance indicates that the anterior vitreous cortex is similar in morphology to the posterior vitreous cortex.^17^ Our results confirm the membranous structures of the anterior vitreous observed in the cadaver eyes using photographic imaging with inorganic salts as contrast agents.^50^

Spatially resolved mapping of light scattered in the anterior vitreous and the lens allowed assessment of relations between these two structures. Hence, we introduced three indices describing the optical properties of the anterior vitreous and the crystalline lens. VOD, VOR, and LOD are based on appropriate processing of the OCT image signal that corresponds to light back-scattered from the anterior vitreous and the crystalline lens. These parameters could be related to previous analyses of vitreous opacities using quantitative ultrasound.^27^ However, the reproducibility of OCT-based parameters outperformed the reproducibility of ultrasound-based quantification, although in both cases 3-D data sets were used for analysis.

In vivo investigation of light scattering in the crystalline lens has previously been conducted using Scheimpflug imaging (densitometry) and OCT and were related to cataract formation and lens aging processes.^51^^–^^54^ More recently, dynamic light scattering was found to be a useful tool to detect the α-crystallin fraction in the lens, which was correlated with the progression of nuclear cataract.^55^^,^^56^ In the present study, the average light intensity scattered in the crystalline lens, as defined by LOD, increased with age with a very strong correlation (R = 0.85), which confirms the well-known effect of age-related loss of lens transparency. Our results also showed a positive correlation between age and the OCT signal in the anterior vitreous.

Anterior vitreous biomarkers (VOD and VOR) were correlated with age. VOD can be interpreted as the average signal from vitreous. In contrast, VOR evaluates vitreous heterogeneity, which is mostly related to the presence of highly scattering opacities. At the same time, VOD and VOR were correlated with the coefficient close to one (R = 0.96), suggesting that despite different underlying methodologies, these parameters can be used interchangeably. We found no significant association between vitreous parameters and axial length, although myopic eyes had higher OSI than nonmyopic eyes (*P* = 0.0121), consistent with previous findings using ultrasound.^9^ This contrasts with a recent study of the central and posterior vitreous in patients with myopic vitreopathy and Vision Degrading Myodesopsia that found greater vitreous echodensity by ultrasound with increasing axial length.^9^ Thus, it may well be that whereas age-related vitreous degeneration affects the entire vitreous body, including the anterior vitreous, myopic vitreopathy spares the anterior vitreous. Future studies should test this hypothesis.

OSI is a biomarker assessing how intra-ocular light scattering affects the retinal PSF. In this study, there was moderate correlation between OSI and VOR or LOD. The mutual contribution of various ocular components to intra-ocular scattering has not been studied, but this result suggests that vitreous may contribute to light scattering considerably and that its smaller reflectivity (relative to the lens) may be offset by a much larger size. Previous studies showed that the patients with vitreous floaters have increased straylight, which is reduced significantly by vitrectomy.^11^ It is not likely that anterior vitreous is important in this regard, because studies showed that preserving the retrolental vitreous during limited vitrectomy nonetheless normalized contrast sensitivity function in 139 cases.^57^ Rather, our striking findings on the quantitative analysis of the anterior vitreous most probably represent an index of concurrent structural abnormalities in the central and posterior vitreous, which are the more likely sources of disturbing light scattering with CSF degradation. On the other hand, VOR contains information on hyperlucent opacification in the anterior vitreous that might be more important for PSF degradation than general transparency of the vitreous body. Future studies that separately evaluate the contributions of different parts of the vitreous body to light scattering should elucidate this issue. To this end, further research should be directed toward development of an OCT system for full vitreous body imaging through compensation of ocular refraction. It is also important to note that OSI is obtained from small angle PSF because the ring between 12 and 20 arcminutes is taken into account while calculating OSI.^44^ It is possible that scatter in the vitreous affects a wider angle of the PSF, which is not included when OSI is calculated.

Several studies have been reported showing the impact of intra-ocular scattering on vision. In particular, recent investigations demonstrated that CSF is degraded by posterior vitreous detachment^12^ and internal vitreous density,^13^^,^^27^ both influenced by myopia and age.^9^^,^^13^ Although OCT-derived parameters introduced in this study can be regarded as descriptors of the optical properties of ocular structures, CSF includes both optical and psychophysical factors in subjective vision performance. Accordingly, there were negative correlations of AULCSF with other parameters (i.e. increased degradation of CSF with increasing vitreous density). This is in agreement with previous studies of cases with Vision Degrading Myodesopsia where echodensity extracted from ultrasound images and Weber CSF were considered.^27^ Their correlation coefficients were, however, higher than obtained in our study, perhaps because those studies imaged the central and posterior vitreous, whereas the present study imaged only the anterior vitreous. Indeed, opacities in the central and posterior vitreous body may be the structures most responsible for retinal image degradation, once again an important topic for future investigation.

Limited field of view was a limitation of this study because there was no mydriasis prior to imaging sessions. Nonetheless, the effective scan captured useful information on the vitreous from the zone inside the pupil (the iris prevents deeper light propagation). This becomes more challenging in older subjects who usually have smaller pupil diameter. In addition, we were not able to identify Wieger's ligament due to the limited field of view.

In conclusion, characterization of microstructural features of the anterior vitreous was enabled by high-resolution 3-D SS-OCT imaging. Age-related opacification of the anterior human vitreous renders it progressively less homogeneous and more light scattering. Quantitative analyses found that vitreous indices associated with the age, but not more robustly than the lens. SS-OCT appears as a useful diagnostic tool for high-resolution optical evaluation of vitreous opacities.

## References

[1] Sebag J. *Vitreous in Health and Disease*. New York, NY: Springer; 2014.

[2] Harocopos GJ, Shui Y-B, McKinnon M, Holekamp NM, Gordon MO, Beebe DC. Importance of vitreous liquefaction in age-related cataract. *Invest Ophthalmol Vis Sci*. 2004; 45: 77–85. 14691157 10.1167/iovs.03-0820

[3] Ankamah E, Sebag J, Ng E, Nolan JM. Vitreous antioxidants, degeneration, and vitreo-retinopathy: exploring the links. *Antioxidants*. 2019; 9: 7. 10.3390/antiox9010007 PMC7022282 31861871

[4] Sebag J. Age-related changes in human vitreous structure. *Graefes Arch Clin Exp Ophthalmol*. 1987; 225: 89–93. 3583000 10.1007/BF02160337

[5] Sebag J. Vitreous anatomy, aging, and anomalous posterior vitreous detachment. In: Dartt DA, Besharse JC, Dana R (eds), *Encyclopedia of the Eye**.* Oxford, UK: Elsevier; 2010: 307–315.

[6] Bishop PN, Holmes DF, Kadler KE, McLeod D, Bos KJ. Age-related changes on the surface of vitreous collagen fibrils. *Invest Ophthalmol Vis Sci*. 2004; 45: 1041–1046. 15037566 10.1167/iovs.03-1017

[7] Ponsioen TL, Hooymans JMM, Los LI. Remodelling of the human vitreous and vitreoretinal interface – a dynamic process. *Prog Ret Eye Res*. 2010; 29: 580–595. 10.1016/j.preteyeres.2010.07.001 20621195

[8] Holekamp NM, Harocopos GJ, Shui Y-B, Beebe DC. Myopia and axial length contribute to vitreous liquefaction and nuclear cataract. *Arch Ophthalmol*. 2008; 126: 744. 18474803 10.1001/archopht.126.5.744-a PMC2585419

[9] Nguyen JH, Nguyen-Cuu J, Mamou J, Routledge B, Yee KMP, Sebag J. Vitreous structure and visual function in myopic vitreopathy causing vision degrading myodesopsia. *Am J Ophthalmol*. 2021; 224: 246–253. 32950508 10.1016/j.ajo.2020.09.017

[10] Milston R, Madigan MC, Sebag J. Vitreous floaters: etiology, diagnostics, and management. *Surv Ophthalmol*. 2016; 61: 211–227. 26679984 10.1016/j.survophthal.2015.11.008

[11] Castilla-Marti M, van den Berg TJ, de Smet MD. Effect of vitreous opacities on straylight measurements. *Retina*. 2015; 35: 1240–1246. 25650709 10.1097/IAE.0000000000000456

[12] Garcia GA, Khoshnevis M, Yee KMP, Nguyen-Cuu J, Nguyen JH, Sebag J. Degradation of contrast sensitivity function following posterior vitreous detachment. *Am J Ophthalmol*. 2016; 172: 7–12. 27633841 10.1016/j.ajo.2016.09.005

[13] Garcia GA, Khoshnevis M, Yee KMP, et al. The effects of aging vitreous on contrast sensitivity function. *Graefes Arch Clin Exp Ophthalmol*. 2018; 256: 919–925. 29536170 10.1007/s00417-018-3957-1

[14] Sebag J. Vitreous and vision degrading myodesopsia. *Prog Ret Eye Res*. 2020; 79: 100847. 10.1016/j.preteyeres.2020.100847 32151758

[15] Sebag J. Diabetic vitreopathy. *Ophthalmology*. 1996; 103: 205–206. 8594502 10.1016/s0161-6420(96)30716-1

[16] Keane PA, Karampelas M, Sim DA, et al. Objective measurement of vitreous inflammation using optical coherence tomography. *Ophthalmology*. 2014; 121: 1706–1714. 24835759 10.1016/j.ophtha.2014.03.006 PMC4507470

[17] Sebag J. Vitreoschisis . *Graefes Arch Clin Exp Ophthalmol*. 2008; 246: 329–332. 18228032 10.1007/s00417-007-0743-x PMC2258312

[18] Sebag J. Anomalous posterior vitreous detachment: a unifying concept in vitreo-retinal disease. *Graefes Arch Clin Exp Ophthalmol*. 2004; 242: 690–698. 15309558 10.1007/s00417-004-0980-1

[19] Kumagai K, Ogino N, Shinjo U, Demizu S, Shioya M, Ueda K. Vitreous opacification after neodymium: YAG posterior capsulotomy. *J Cataract Refract Surg*. 1999; 25: 981–984. 10404376 10.1016/s0886-3350(99)00087-5

[20] Tanaka H, Ohara K, Shiwa T, Minami M. Idiopathic opacification of Berger's space. *J Cataract Refract Surg*. 2004; 30: 2232–2234. 15474843 10.1016/j.jcrs.2004.05.014

[21] Mares V, Nehemy MB, Salomão DR, Goddard S, Tesmer J, Pulido JS. Multimodal imaging and histopathological evaluation of Berger's space. *Ocul Oncol Pathol*. 2020; 6: 3–9. 32002397 10.1159/000495724 PMC6984151

[22] Gonzalez R, Cheng H, Barnett P, et al. Nuclear magnetic resonance imaging of the vitreous body. *Science*. 1984; 223: 399–400. 6318321 10.1126/science.6318321

[23] Walton KA, Meyer CH, Harkrider CJ, Cox TA, Toth CA. Age-related changes in vitreous mobility as measured by video B scan ultrasound. *Exp Eye Res*. 2002; 74: 173–180. 11950227 10.1006/exer.2001.1136

[24] Sebag J. Imaging vitreous. *Eye*. 2002; 16: 429–439. 12101450 10.1038/sj.eye.6700201

[25] Sebag J. Seeing the invisible: the challenge of imaging vitreous. *J Biomed Opt*. 2004; 9: 38–46. 14715056 10.1117/1.1627339

[26] Restori M. Imaging the vitreous: optical coherence tomography and ultrasound imaging. *Eye*. 2008; 22: 1251–1256. 18292783 10.1038/eye.2008.30

[27] Mamou J, Wa CA, Yee KMP, et al. Ultrasound-based quantification of vitreous floaters correlates with contrast sensitivity and quality of life. *Invest Ophthalmol Vis Sci*. 2015; 56: 1611–1617. 25613948 10.1167/iovs.14-15414 PMC4554261

[28] Azhdam AM, Goldberg RA, Ugradar S. In vivo measurement of the human vitreous chamber volume using computed tomography imaging of 100 eyes. *Transl Vis Sci Technol*. 2020; 9: 2. 10.1167/tvst.9.1.2 PMC7255624 32509437

[29] Fujimoto JG, Drexler W. (eds). *Optical Coherence Tomography. Technology and Applications*. New York, NY: Springer; 2015.

[30] Mojana F, Kozak I, Oster SF, et al. Observations by spectral-domain optical coherence tomography combined with simultaneous scanning laser ophthalmoscopy: imaging of the vitreous. *Am J Ophthalmol*. 2010; 149: 641–650. 20138610 10.1016/j.ajo.2009.11.016

[31] Muqit MMK, Stanga PE. Swept-source optical coherence tomography imaging of the cortical vitreous and the vitreoretinal interface in proliferative diabetic retinopathy: assessment of vitreoschisis, neovascularisation and the internal limiting membrane. *Br J Ophthalmol*. 2014; 98: 994–997. 24659354 10.1136/bjophthalmol-2013-304452

[32] Tsukahara M, Mori K, Gehlbach PL, Mori K. Posterior vitreous detachment as observed by wide-angle OCT imaging. *Ophthalmology*. 2018; 125: 1372–1383. 29631900 10.1016/j.ophtha.2018.02.039

[33] Liu JJ, Witkin AJ, Adhi M, et al. Enhanced vitreous imaging in healthy eyes using swept source optical coherence tomography. *PLoS One*. 2014; 9: e102950. 25036044 10.1371/journal.pone.0102950 PMC4103882

[34] Pang CE, Freund KB, Engelbert M. Enhanced vitreous imaging technique with spectral-domain optical coherence tomography for evaluation of posterior vitreous detachment. *JAMA Ophthalmol*. 2014; 132: 1148–1150. 25010436 10.1001/jamaophthalmol.2014.1037

[35] Stanga P, Sala-Puigdollers A, Caputo S, et al. In vivo imaging of cortical vitreous using 1050-nm swept-source deep range imaging optical coherence tomography. *Am J Ophthalmol*. 2014; 157: 397–404.e392. 24439443 10.1016/j.ajo.2013.10.008

[36] Song M, Shen M, Zhou Y, et al. Observation of vitreous features using enhanced vitreous imaging optical coherence tomography in highly myopic retinoschisis. *Retina*. 2019; 39: 1732–1741. 29912094 10.1097/IAE.0000000000002226

[37] Hua R, Ning H. Modified enhanced vitreous imaging modality of spectral domain optic coherence tomography. *Eye*. 2021; 35: 351–352. 32055017 10.1038/s41433-020-0814-3 PMC7852669

[38] Grulkowski I, Liu JJ, Potsaid B, et al. Retinal, anterior segment and full eye imaging using ultrahigh speed swept source OCT with vertical-cavity surface emitting lasers. *Biomed Opt Express*. 2012; 3: 2733–2751. 23162712 10.1364/BOE.3.002733 PMC3493240

[39] Menapace R. Transzonular capsulo-hyaloidal hydroseparation with optional triamcinolone enhancement: a technique to detect or induce anterior hyaloid membrane detachment for primary posterior laser capsulotomy. *J Cataract Refract Surg*. 2019; 45: 903–909. 31262480 10.1016/j.jcrs.2019.03.008

[40] Santos-Bueso E. Berger's space. *Arch Soc Esp Oftalmol*. 2019; 94: 471–477. 31378388 10.1016/j.oftal.2019.06.006

[41] Otero C, Vilaseca M, Arjona M, Martínez-Roda J, Pujol J. Repeatability of aberrometric measurements with a new instrument for vision analysis based on adaptive optics. *J Refract Surg*. 2015; 31: 188–194. 25751836 10.3928/1081597X-20150224-03

[42] Hervella L, Villegas EA, Prieto PM, Artal P. Assessment of subjective refraction with a clinical adaptive optics visual simulator. *J Cataract Refract Surg*. 2019; 45: 87–93. 30309774 10.1016/j.jcrs.2018.08.022 PMC6320260

[43] Güell JL, Pujol J, Arjona M, Diaz-Douton F, Artal P. Optical quality analysis system: instrument for objective clinical evaluation of ocular optical quality. *J Cataract Refract Surg*. 2004; 30: 1598–1599. 15210251 10.1016/j.jcrs.2004.04.031

[44] Artal P, Benito A, Pérez GM, et al. An objective scatter index based on double-pass retinal images of a point source to classify cataracts. *PLoS One*. 2011; 6: e16823. 21326868 10.1371/journal.pone.0016823 PMC3033912

[45] Grulkowski I, Manzanera S, Cwiklinski L, et al. Volumetric macro- and micro-scale assessment of crystalline lens opacities in cataract patients using long-depth-range swept source optical coherence tomography. *Biomed Opt Express*. 2018; 9: 3821–3833. 30338158 10.1364/BOE.9.003821 PMC6191641

[46] Spaide RF, Koizumi H, Pozonni MC. Enhanced depth imaging spectral-domain optical coherence tomography. *Am J Ophthalmol*. 2008; 146: 496–500. 18639219 10.1016/j.ajo.2008.05.032

[47] Pérez GM, Manzanera S, Artal P. Impact of scattering and spherical aberration in contrast sensitivity. *J Vis*. 2009; 9: 19. 10.1167/9.3.19 19757958

[48] Hwang ES, Kraker JA, Griffin KJ, Sebag J, Weinberg DV, Kim JE. Accuracy of spectral-domain OCT of the macula for detection of complete posterior vitreous detachment. *Ophthalmol Retina*. 2020; 4: 148–153. 31864940 10.1016/j.oret.2019.10.013 PMC7008078

[49] Grulkowski I, Manzanera S, Cwiklinski L, Sobczuk F, Karnowski K, Artal P. Swept source optical coherence tomography and tunable lens technology for comprehensive imaging and biometry of the whole eye. *Optica*. 2018; 5: 52–59.

[50] Kislitsyna NM, Novikov SV, Kolesnik SV, Veselkova MP, Dibirova S. Anatomic-topographic features of the anterior cortical layers of the vitreous body. *EC Ophthalmol*. 2019; 10: 1–10.

[51] Alió JL, Schimchak P, Negri HP, Montés-Micó R. Crystalline lens optical dysfunction through aging. *Ophthalmology*. 2005; 112: 2022–2029. 16183126 10.1016/j.ophtha.2005.04.034

[52] Grewal DS, Brar GS, Grewal SPS. Correlation of nuclear cataract lens density using Scheimpflug images with lens opacities classification system III and visual function. *Ophthalmology*. 2009; 116: 1436–1443. 19500847 10.1016/j.ophtha.2009.03.002

[53] Lim SA, Hwang J, Hwang K-Y, Chung S-H. Objective assessment of nuclear cataract: comparison of double-pass and Scheimpflug systems. *J Cataract Refract Surg*. 2014; 40: 716–721. 24767907 10.1016/j.jcrs.2013.10.032

[54] de Castro A, Benito A, Manzanera S, et al. Three-dimensional cataract crystalline lens imaging with swept-source optical coherence tomography. *Invest Ophthalmol Vis Sci*. 2018; 59: 897–903. 29435589 10.1167/iovs.17-23596

[55] Ansari RR, Suh KI, Dunker S, Kitaya N, Sebag J. Quantitative molecular characterization of bovine vitreous and lens with non-invasive dynamic light scattering. *Exp Eye Res*. 2001; 73: 859–866. 11846516 10.1006/exer.2001.1097

[56] Datiles MB 3rd, Ansari RR, Yoshida J, et al. Longitudinal study of age-related cataract using dynamic light scattering: loss of α-crystallin leads to nuclear cataract development. *Ophthalmology*. 2016; 123: 248–254. 26545319 10.1016/j.ophtha.2015.10.007 PMC4724511

[57] Sebag J, Yee KMP, Nguyen JH, Nguyen-Cuu J. Long-term safety and efficacy of limited vitrectomy for vision degrading myodesopsia resulting from vitreous floaters. *Ophthalmol Retina*. 2018; 2: 881–887. 31047219 10.1016/j.oret.2018.03.011

